# A machine learning approach for modeling decisions in the out of hospital cardiac arrest care workflow

**DOI:** 10.1186/s12911-021-01730-4

**Published:** 2022-01-25

**Authors:** Samuel Harford, Marina Del Rios, Sara Heinert, Joseph Weber, Eddie Markul, Katie Tataris, Teri Campbell, Terry Vanden Hoek, Houshang Darabi

**Affiliations:** 1 Department of Mechanical and Industrial Engineering, University of Illinois at Chicago, Chicago, IL USA; 2 Department of Emergency Medicine, University of Iowa – Carver College of Medicine, Iowa City, IA USA; 3 Department of Emergency Medicine, Rutgers Robert Wood Johnson Medical School, New Brunswick, NJ USA; 4 Department of Emergency Medicine, John H. Stroger, Jr. Hospital, Chicago, IL USA; 5 Illinois Masonic Medical Center, Chicago, IL USA; 6 Department of Emergency Medicine, University of Chicago, Chicago, IL USA; 7 Department of Emergency Medicine, University of Illinois at Chicago, Chicago, IL USA

**Keywords:** Out of hospital cardiac arrest, Neurological outcome, Decision modeling, Machine learning, Deep learning

## Abstract

**Background:**

A growing body of research has shown that machine learning (ML) can be a useful tool to predict how different variable combinations affect out-of-hospital cardiac arrest (OHCA) survival outcomes. However, there remain significant research gaps on the utilization of ML models for decision-making and their impact on survival outcomes. The purpose of this study was to develop ML models that effectively predict hospital’s practice to perform coronary angiography (CA) in adult patients after OHCA and subsequent neurologic outcomes.

**Methods:**

We utilized all (N = 2398) patients treated by the Chicago Fire Department Emergency Medical Services included in the Cardiac Arrest Registry to Enhance Survival (CARES) between 2013 and 2018 who survived to hospital admission to develop, test, and analyze ML models for decisions after return of spontaneous circulation (ROSC) and patient survival. ML classification models, including the Embedded Fully Convolutional Network (EFCN) model, were compared based on their ability to predict post-ROSC decisions and survival.

**Results:**

The EFCN classification model achieved the best results across tested ML algorithms. The area under the receiver operating characteristic curve (AUROC) for CA and Survival were 0.908 and 0.896 respectively. Through cohort analyses, our model predicts that 18.3% (CI 16.4–20.2) of patients should receive a CA that did not originally, and 30.1% (CI 28.5–31.7) of these would experience improved survival outcomes.

**Conclusion:**

ML modeling effectively predicted hospital decisions and neurologic outcomes. ML modeling may serve as a quality improvement tool to inform system level OHCA policies and treatment protocols.

**Supplementary Information:**

The online version contains supplementary material available at 10.1186/s12911-021-01730-4.

## Introduction

Out-of-hospital cardiac arrest (OHCA) is a critical public health burden affecting approximately 400,000 persons in the United States annually where only 10% survive [1]. While advances in resuscitation science have improved survival rates, mortality varies widely by geography, emergency medical services (EMS) agency, and hospital [2]. While some of the variation has been attributed to OHCA characteristics (i.e., presenting rhythm, age, receipt of bystander cardiopulmonary resuscitation), variations in post-cardiac-arrest hospital care, such as use of coronary angiography and revascularization, when needed, may explain some of the heterogeneity seen when comparing survival and good neurological outcome [3].

Machine Learning (ML) is a subfield of artificial Intelligence (AI) where algorithms learn tasks by studying high volumes of data [4, 5]. The advent of big data and use of electronic health records enable us to pursue solutions to critical health issues. While traditional statistical methods are the standard for investigating patient and treatment intervention and associated outcomes, studies have suggested that ML algorithms provide greater insights across a wide variety of clinical settings [6]. AI models use data to predict future events on the basis of the statistical weight of historical correlations and identify sensitive points within the system of care to direct strategic allocation of resources to improve disparities in clinical outcomes. ML has already proved useful in healthcare applications including medical imaging [7], disease outbreak prediction [8], drug discovery/usage [9, 10], and hospital workflow optimization [11, 12].

The OHCA care workflow is a time-sensitive process that requires quick and effective decision making throughout the chain of survival. ML has been applied at several stages of the care workflow to aid in predicting risk and recognition of cardiac arrest. During calls to emergency centers, conversations can be monitored using a ML model to help identify a cardiac arrest [13]. Wearable devices can monitor vitals to predict the occurrence of a cardiac arrest for high risk individuals [14]. ML has also been used to predict in-hospital cardiac arrests (IHCA) based on vital monitoring [15].

Previous ML studies in OHCA have been limited by small population size and lack of diversity [16], absence of pre-hospital data in model development [17], and by not discriminating overall survival from survival with good neurologic outcomes [17]. In addition, there remain significant research gaps on the utilization of ML models for post-return of spontaneous circulation (ROSC) decision-making throughout the OHCA workflow and their impact on survival outcomes. Powerful and affordable computer technologies enable us to combine big data to evaluate interactions that affect decision-making and survival. This study aims to develop ML models that effectively predict hospital’s post-ROSC practice to perform coronary angiography in adult patients with ROSC after OHCA and subsequent neurologic outcomes.

## Methods

This study was approved by the Office for the Protection of Research Subjects of the University of Illinois at Chicago.

### Study setting

The Chicago Fire Department (CFD) is the sole EMS agency providing emergency response and transport for 911 calls for Chicago’s approximately three million residents. Upon identification of an OHCA by EMS dispatchers, a basic or advanced life support fire suppression company, and advanced life support transport ambulance are dispatched to the cardiac arrest. The Chicago EMS system responds to over 2500 OHCA incidents annually and has 33 receiving hospitals for OHCA incidents, including 24 ST-elevation myocardial infarction (STEMI) hospitals with interventional cardiology and targeted temperature management capabilities [18]. Per protocol, all OHCA patients treated by CFD EMS who either achieve ROSC or with refractory ventricular fibrillation or ventricular tachycardia are transported to STEMI receiving centers in order to ensure access to early coronary angiography and revascularization as these are a critical component of post-resuscitation care [19].

### Data collection

This study utilizes Cardiac Arrest Registry to Enhance Survival (CARES) data for Chicago [20]. The CARES registry is the largest cardiac arrest data source in the United States, collecting 911 dispatch centers, EMS providers, and receiving hospital data from over 1800 EMS agencies and 2200 hospitals [21].

**Fig. 1:**
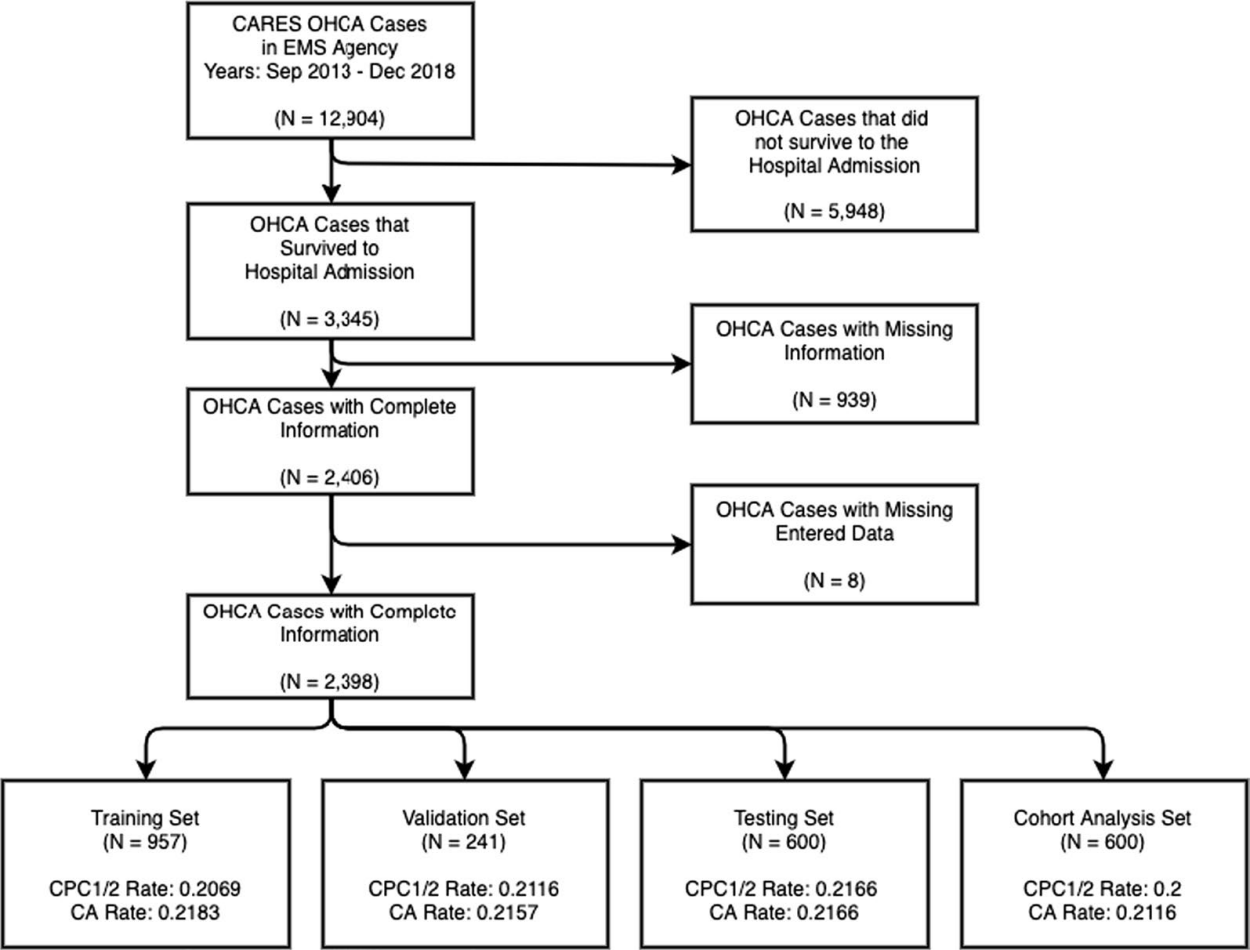
Data flow for machine learning modeling subsets

The CFD EMS treated 12,904 non-traumatic OHCA incidents between September 2013 and December 2018. This study focuses on OHCA workflow decisions made post-ROSC including 2398 OHCA incidents that survived to hospital admission. Figure 1 shows how the data is preprocessed for the study from the full Chicago CARES data. The information used for modeling includes 23 input features for decision modeling and 24 input features for survival modeling (see Additional file 1: Table S2 for detailed lists of the decisions and outcomes in this data source). During the modeling process for the decision and survival models, the information has a sequential nature where the only new piece of information in the survival model is the Coronary Angiography. The input features used to develop and evaluate models are all categorical features. To accommodate ML models, the categorical features are either one-hot encoded or transformed into a vectorized representation [22]. Traditional forms of machine learning algorithms require categorical features to be one-hot encoded where a single feature is transformed into multiple features that represent the original information. For example, if the feature regarding the initiation of cardiopulmonary resuscitation (CPR) has the options of Lay Person, First Responder, or EMS Personnel and the instance receives First Responder CPR the value is encoded as [0,1,0]. In the neural network models, certain architectures allow for the utilization of embedding layers. These embedding layers allow for the model to learn a vectorized representation of the features instead of going through the one-hot encoding process.

This study models post-ROSC decisions and the neurologic outcome of OHCA incidents. CARES includes data on two post-ROSC decisions: (1) whether a patient underwent targeted temperature management (TTM) and (2) whether coronary angiography (CA) was performed. The 2015 AHA Guidelines recommended (Class I) that comatose adult patients with ROSC after OHCA have TTM and CA for OHCA patients with suspected cardiac etiology of arrest and ST-elevation on ECG (Class I). The 2015 AHA Guidelines also stated that CA is reasonable even in the absence of ST-elevation (Class IIa) as CA, alone or as part of a bundle of care, is associated with improved myocardial function and neurological outcomes [23–25]. Due to wide variation in TTM practice and absence of granular data regarding individual hospital practices (e.g., temperature selected, length of temperature management, rewarming practices), we did not consider the decision to perform TTM as one that could be reliably predicted in decision models. Instead we opted for the post-ROSC decision of CA, where the modeling process aims to determine whether or not a patient is expected to receive a Coronary Angiography. The CA model refers to the decision model throughout this paper.

We also examine the outcome of neurologic function from the hospital record, measured in CARES by Cerebral Performance Category (CPC) score [26]. For this study, neurologic outcome was modified to a binary classification based on CPC score: Class 0 of individuals who survived with functional neurological outcomes (CPC1/2) and Class 1 of patients with non-functional neurological outcomes (CPC3/4/5). The CPC model aims to classify patients into one of these classes based on the care and condition of the patient. The CPC model refers to the survival model throughout this paper.

### Machine learning modeling

To classify the OHCA workflow decisions and neurological outcome, we train ML models using a subset of the available data. We then evaluate the performance of the developed model on the remaining data, i.e. data not used in the training phase. For the 2398 OHCA instances in the study, 21.5% receive a Coronary Angiography, and 20.8% have a neurological outcome of Class 0. Figure 1 subsets the 2398 OHCA instances for this study into four sets: training, validation, testing, and cohort analysis. The modeling sets are split randomly across the data timeframe because the decision-making policies implemented during the timeframe should be consistent. The training set consists of 957 events (40% of data), and is used to construct the ML models. The validation set consists of 241 events (10% of data), and is used for model parameter optimization and model comparison. The testing set consists of 600 events (25% of data), and is used to evaluate models on completely unseen data. The cohort analysis set consists of 600 events (25% of data), and is used to perform further analysis regarding post-ROSC decisions for cohorts that will be formally defined in the Cohort Analysis subsection. Additional file 1: Table S1 provides additional details for the data demographics for each set.

To model the decisions and survival outcomes we compare several ML algorithms. LightGBM [27], XGBoost [28], Decision Trees [29], Random Forest [30], Gradient Boosting [31], k-Nearest Neighbor [32], Logistic Regression [33], Support Vector Machine [34], and Deep Neural Networks [35]. ML models including Logistic Regression, Decision Trees, and k-Nearest Neighbor are popular algorithms in a variety of healthcare applications because they are highly interpretable to the user. The remaining developed ML models are generally more powerful algorithms, however they result in low interpretable outcomes. During the training process for all models a grid search of parameters performed to ensure that each model is optimized. Additional file 1: Table S3 provides the detailed parameters that are explored during this process.

**Fig. 2:**
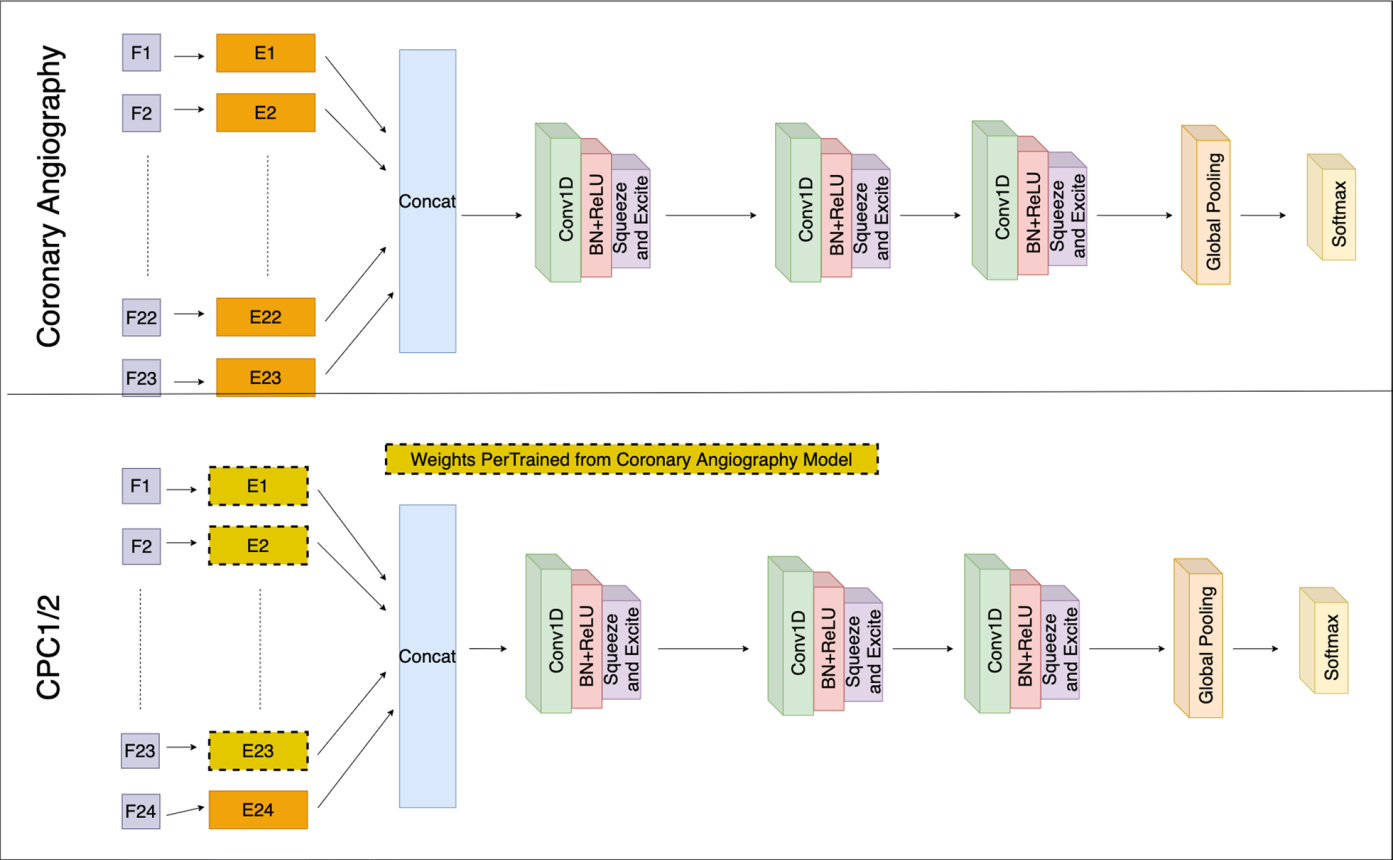
Sequence of EFCN architectures with transfer learning

For the neural network model, we utilized a modified Embedded Fully Convolutional Network (EFCN) [36]. Figure 2 illustrates the EFCN model architecture for the two types of models: Coronary Angiography and CPC Score. These two models are developed in a sequence where the Coronary Angiography EFCN is trained first, then the CPC Score EFCN. Due to the sequential nature of the modeling flow and the utilization of neural networks, transfer learning is applied by using pre-trained embedding vectors [37]. As shown in Fig. 2, the embedding weights for the Coronary Angiography EFCN model are randomly initialized and updated during training. For the CPC Score EFCN model, the pre-trained embedding vectors (yellow dashed boxes) are used as the initial weights for the embedding vectors and then updated during the training from the Coronary Angiography classification. The only randomly initialized weights for the CPC Score EFCN model is the embedding vector for the Coronary Angiography input, which is the only new feature in this model.

Each model is constructed using the training set and was iteratively improved using the validation set. The model that yields the best area under the receiver operating characteristic curve (AUROC) on the validation data is selected as the best model. The higher the AUROC, the better the performance of the model at distinguishing between classes. This model is then evaluated on the unseen testing data. AUROC is used as the primary evaluation metric. In addition, other metrics including the accuracy, area under the precision-recall curve (AUPRC), F1 Score, Matthew’s correlation coefficient (MCC) [38], and the Brier score loss (BSL) [39] are reported for all results. In addition, the SHapley Additive exPlanations (SHAP) [40] values for the best method is present. This method utilizes game theory to explain the prediction of an instance by calculating the contribution of each feature to the final prediction.

### Cohort analysis

Chicago has 33 receiving hospitals. Using the developed ML models, cohort analysis of post-ROSC decisions was performed. Prior to analysis, the hospitals first are broken into cohorts based on the CPC1/2 rates for applicable OHCA instances. This analysis has the potential to be conducted on all hospitals, but to ensure generalizability we removed 18 hospitals from this analysis due to an insufficient number of OHCA instances to draw conclusions about the post-ROSC decisions. The cohort analysis data set includes 15 hospitals with at least 75 OHCA instances for the study period. The 15 hospitals are broken into three cohorts with five hospitals in each cohort. The CPC1/2 rate of each cohort is determined based on the combined CPC1/2 rates of the training and validation instances of the hospitals in the respective cohort. Cohort 1 consists of the 5 hospitals with the highest CPC1/2 rate of 35.9. Cohort 2 consists of the 5 hospitals with the next highest CPC1/2 rate of 18.3. Cohort 3 consists of the remaining 5 hospitals with a CPC1/2 rate of 13.2. Additional file 1: Table S1 provides a demographic breakdown of these cohorts.

The cohorts were used to retrain our ML models to evaluate how decisions differ based on hospital cohorts. Modeling retraining is done by taking the pretrained weights from the full modeling process and allows the model to reoptimize because on just a specific cohorts training set. The retraining process allows for the models to better reflect a cohorts decision making without started from randomized network weights. These cohort models are used to analyze how different cohorts make decisions when patients arrive with specific field conditions. The potential field conditions for analysis include the field data listed in Additional file 1:Table S2.

## Results

The training set consists of 957 OHCA patients. Of these patients, 209 receive a Coronary Angiography, and 198 have a neurological outcome of Class 0. The training set is used to develop the ML models. Each model utilizes the training data to learn the underlying patterns in the data with the objective of performing the classification task. For the decision making model, the objective is to learn how the decision to perform a Coronary Angiography is being made based on the real instances recorded in the data.

The validation set consists of 241 cardiac arrest patients, of which, 52 receive a Coronary Angiography, and 51 have a neurological outcome of Class 0. Additional file 1: Table S3 provides detailed information about the optimized hyperparameters for each model. Table 1 presents the AUROCs for each ML model on the tasks of modeling CA and CPC. The EFCN model achieves the best AUROC for CA and CPC with scores of 0.8836 and 0.9272, respectively. Additional file 1: Table S4 provides complete modeling results of all models with a variety of metrics and complete confusion matrices.

**Table 1:** Results on validation set in terms of AUROC

Model	CA	CPC
LightGBM	0.7050	0.7462
Random Forest	0.6641	0.6437
XGBoost	0.6639	0.7561
Gradient Boost	0.6619	0.6977
Decision Tree	0.6415	0.6537
k-Nearest Neighbor	0.6781	0.7077
Logistic Regression	0.6937	0.7417
EFCN	0.8836	0.9272

The testing set consists of 600 cardiac arrest patients, of which 130 receive a CA, and 130 have a neurological outcome of Class 0. The AUROC of the CA and CPC EFCN models on the testing set are 0.9079 and 0.8967, respectively. Additional file 1: Table S2 provides SHAP value averages for each model broken by class. Additional file 1: Table S4 provides information about the testing results of all models and additional evaluation metrics.

The cohort analysis set consists of 600 cardiac arrest patients. For these 600, 94 are removed in unused hospitals as discussed in the Cohort Analysis subsection. For the cohorts there are 132, 156, and 218 cardiac arrest patients in Cohorts 1–3, respectively. Each of these cohorts have 26, 34, and 43 patients that receive a CA, respectively. The CA EFCN model is reoptimized based on the Cohort 1 training and validation data. When evaluating the performance of the reoptimized model on the respective cohort sets, AUROCs of 0.9761, 0.6601, and 0.6371 are achieved for the respective cohorts. Table 2 demonstrates the expected model changes of the reoptimized Cohort 1 model on the other 2 cohorts. This table first shows the patients that did not receive a Coronary Angiography and then what happens to their expected output where a positive change means that a patient is now expected to survive with CPC1 or 2. The Cohort 1 model expected 33 of the 175 Cohort 3 patients without a CA to be given a CA. Then using the survival model, the Cohort 1 model predicts a positive change in survival for 10 of the 33 patients with a changed CA.

**Table 2:** Cohort analysis of the reoptimized Cohort 1 EFCN models on the other cohort data

Cohort 2 (N = 156)		Cohort 3 (N = 218)	
Patients that were not initially given CA, that the model predicts to get CA *23/122* *(18.85%)*	No change in CPC class *18/23 (78.3%)*	Patients that were not initially given CA, that the model predicts to get CA *33/175* *(18.86%)*	No change in CPC class *23/33 (69.7%)*
	Positive change in CPC class *5/23 (21.7%)*		Positive change in CPC class *10/33 (30.3%)*
	Negative change in CPC class *0/23 (0%)*		Negative change in CPC class *0/33 (0%)*

## Discussion

Our decision and survival models achieve testing AUROCs of 0.9079 and 0.8967, respectively. These evaluation metrics are similar to the AUROCs of the validation set, which suggests these models do well in generalizing to unseen data. Our cohort analysis showed how modeled changes in decisions could impact OHCA survival. When evaluating the lowest tertile (Cohort 3) with models based on the highest tertile (Cohort 1), our model showed a change in the decision to perform CA for 18.86% of the patients and predicted a positive impact in CPC class for 30.3% of the patients with a changed decision. To our knowledge, this is the first study to show that ML modeling can not only effectively predict patient outcome after an OHCA but can also predict hospital practice to perform CA post-ROSC.

Because decision-making in healthcare often involves large amounts of data, ML and simulation can be useful tools to predict how different variable combinations affect patient outcomes [6]. ML has been successfully used in prognosis, diagnosis, treatment, clinical workflow, and expanding the availability of clinical expertise [41]. In our previous work, a ML model using data from the Chicago CARES dataset had an AUROC of 0.825 in predicting survival with favorable neurological outcomes among patients with a witnessed OHCA [36]. A Korean study of deep learning ML models used electronic health record data to predict subsequent cardiac arrest in hospitalized patients with an AUROC of 0.850 [15]. ML models of OHCA can also predict survival outcome. A study from the Swedish Registry of Cardiopulmonary Resuscitation (SRCR) reported an accuracy of 0.82 in predicting survival after OHCA [42]. The Korean OHCA registry had a better performance in predicting neurologic outcomes with an AUROC of 0.953, but this study included only patients who had sustained ROSC [43]. In our study utilizing data from patients who survived to hospitalization post-OHCA, the AUROC of 0.8967 for survival with functional CPC was better than both previous studies.

Perhaps the most powerful use of ML models is as virtual laboratories for examining the interaction of treatment strategies and interventions under different patient variables and systems of care circumstances that may be otherwise costly, time-consuming, or even unethical to manipulate in the real world. Such models can help decision-makers within OHCA systems of care make tactical decisions regarding resource allocation or adapt treatment guidelines to local context. Ours is the first model to predict decisions with a high accuracy level regarding provision of CA after OHCA with an AUROC 0.8836. Moreover, we were able to show that when hospitals in Cohort 3 (lowest CPC tertile) changed the decision-making regarding coronary angiography to resemble decisions made by Cohort 1 (highest CPC tertile), more patients could survive with functional neurologic outcome. OHCA systems of care can use ML models to critically review OHCA treatment guidelines and test how different decisions may affect patient outcomes before costly and time-consuming implementation and training.

Our findings also have significant implications for how emergency systems of care define cardiac arrest care centers (CACs). Some studies have suggested that hospital case volume and coronary angiography capabilities are associated with better outcomes [44–47]. However, post-OHCA care is complex and requires the coordination of multiple specialties including neurointensivists, cardiologists, pulmonary, and critical care specialists, to name a few. Our study demonstrates the power of ML as a tool to inform decision-making for systems of care. In the future, EMS systems of care without formalized CAC agreements could develop ML models to identify which hospitals to preferentially transport patients post-OHCA. ML models can also be developed to perform continuous quality improvement of treatment in the field and on the hospital side of care. Hospitals can also use ML for benchmarking against other hospitals within the same system of care and to simulate how changes in local treatment guidelines impact patient outcomes before implementing these changes at a larger scale. These ML models can also be adapted to other emergency systems of care such as stroke, myocardial infarction, and trauma.

One of our study limitations is its limited generalizability as external validation is needed to further interrogate the performance of the final model. Another important limitation is our inability to define why hospitals make different interventions decisions although caring for patients with similar demographics and cardiac arrest characteristics. Specifically, the CARES data does not include data on the presence or absence of ST-elevation on EKG and it does not provide sufficient detail to measure and compare the utilization of other resources, such as expertise in neuroprognostication or the presence of a cardiac arrest champion. The CARES data set is also limited in that it does not include details on comorbid illnesses that influence prognostication and the decision to perform CA such as cancer and end stage renal disease. Despite the data limitations, our models show promise for ML as a tool to predict hospital variations in post-ROSC care and subsequent neurologic outcome in OHCA.

## Conclusion

Our study showed a modeled difference in a decision to perform coronary angiography between hospitals by tertile for survival with CPC 1–2. Artificial intelligence and ML can be a valuable tool to guide systems of care decision-making. Future research may develop a more reliable decision support network by incorporating more detailed individual patient-level and system level features.

## Supplementary Information

**Additional file 1. Table S1.** Patient Demographics as a Percent of the Dataset. **Table S2**: Input Features for Each Model and a Feature Importance Representation of SHAP Values for the testing set utilizing the EFCN Model. **Table S3**: Detailed Parameters for Each Model. **Table S4**: Detailed modeling results on both the validation and testing sets.

## Data Availability

The data that support the findings of this study are available from the Cardiac Arrest Registry to Enhance Survival (CARES) but restrictions apply to the availability of these data, which were used under license for the current study, and so are not publicly available. Data are however available from the authors upon reasonable request and with permission of CARES data sharing committee.

## Abbreviations

AUROC: Area under the receiver operating characteristic
CA: Coronary angiography
CARES: Cardiac arrest registry to enhance survival
CFD: Chicago fire department
CPC: Cerebral performance category
EFCN: Embedded fully convolutional network
EMS: Emergency medical services
ML: Machine learning
OHCA: Out-of-hospital cardiac arrest
ROSC: Return of spontaneous circulation
TTM: Targeted temperature management
