# Supplementary material for: A machine learning approach for modeling decisions in the out of hospital cardiac arrest care workflow
Source: BMC Med Inform Decis Mak. 2022 Jan 25;22:21. doi: 10.1186/s12911-021-01730-4 (PMC8787933; doi:10.1186/s12911-021-01730-4)
Supplement: Supplementary file 1 — Additional file 1. Table S1. Patient Demographics as a Percent of the Dataset. Table S2: Input Features for Each Model and a Feature Importance Representation of SHAP Values for the testing set utilizing the EFCN Model. Table S3: Detailed Parameters for Each Model. Table S4: Detailed modeling results on both the validation and testing sets. [file 12911_2021_1730_MOESM1_ESM.docx]

**Supplementary Tables**

Table S1. Patient Demographics as a Percent of the Dataset

| Patient and Arrest Characteristics | | Training  (N = 957) | Validation  (N = 241) | Testing  (N =600) | Sensitivity  (N=600)  [Demographics Only shown for the cohort patients, if patients are removed from study they are not included in demographic breakdown] | | |
| --- | --- | --- | --- | --- | --- | --- | --- |
|  |  |  |  |  | Cohort 1  (N=132) | Cohort 2  (N=156) | Cohort 3  (N=218) |
| Gender | Male | 55.6 | 59.3 | 58 | 50 | 51.9 | 54.2 |
| Ethnicity | Asian | 2.61 | 1.66 | 2.83 | 1.52 | 1.92 | 0.4 |
|  | Black/AA | 50.9 | 53.5 | 52.5 | 38.6 | 53.2 | 64.3 |
|  | Hispanic/  Latino | 12.2 | 11.6 | 13.8 | 15.2 | 12.2 | 11.1 |
|  | White | 31.5 | 30.7 | 28.8 | 43.2 | 30.1 | 20.5 |
| Age | <40 | 10.6 | 8.57 | 7.42 | 7.8 | 7.1 | 6.5 |
|  | 40-59 | 31.57 | 26.44 | 27.26 | 31.9 | 28.4 | 26.7 |
|  | 60-75 | 32.73 | 36.55 | 38.61 | 34.5 | 36.2 | 37.6 |
|  | >75 | 25.1 | 28.44 | 26.71 | 25.8 | 28.3 | 29.2 |
| First Rhythm Type | Shockable | 24.2 | 29.4 | 27.8 | 30.1 | 29.5 | 28.9 |
| Witness Status | Witnessed | 61.7 | 62.8 | 62.8 | 62.8 | 62.2 | 61.5 |
| CPR Initiation | First Responder | 34.7 | 35.1 | 35.6 | 34.6 | 36.2 | 34.5 |
|  | EMS Personal | 42.7 | 42.4 | 42.1 | 42.5 | 41.7 | 43.2 |
|  | Lay Person  (Any) | 22.6 | 22.5 | 22.3 | 22.9 | 22.1 | 22.3 |
| AED Application | First Responder | 13.1 | 13.5 | 13.3 | 14.7 | 13.4 | 13.2 |
|  | Lay Person | 4.5 | 4.3 | 4.2 | 5.1 | 4.3 | 4.0 |

Table S2: Input Features for Each Model and a Feature Importance Representation of SHAP Values for the testing set utilizing the EFCN Model.

| **Data Feature** | **CA** | | | **CPC** | | |
| --- | --- | --- | --- | --- | --- | --- |
|  | **Feature Utilized** | **SHAP Average**  **Class No** | **SHAP Average Class Yes** | **Feature Utilized** | **SHAP Average**  **Class 0** | **SHAP Average Class 1** |
| 1. Age (4 Categories) | *X* | *-0.000234* | *-0.00278* | *X* | *0.000342* | *-0.000483* |
| 1. Incident Zip Code | *X* | -0.0001154 | -0.0028107 | *X* | 0.00154175 | -0.0001598 |
| 1. Gender | *X* | 0.00091887 | -0.0017429 | *X* | 0.00095515 | -0.0010339 |
| 1. Race/Ethnicity | *X* | 0.000789 | *-0.000232* | *X* | *0.0002837* | *-0.000282* |
| 1. First Responder | *X* | 0.00045187 | -0.0014072 | *X* | 0.00183462 | -0.0004193 |
| 1. Destination Hospital | *X* | 0.00070676 | -0.0109299 | *X* | 0.00623883 | -0.0005443 |
| 1. Location Type | *X* | 0.00057707 | -0.0011332 | *X* | 0.00081689 | -0.0002368 |
| 1. Arrest Witness Status | *X* | 0.00111486 | -0.0037415 | *X* | -0.0001848 | -0.000156 |
| 1. Presumed Cardiac Arrest Etiology | *X* | 0.00036069 | -0.0003996 | *X* | 0.00064902 | -0.0006274 |
| 1. Initiated CPR | *X* | 0.00046419 | -0.0032791 | *X* | 0.00560737 | -0.0016027 |
| 1. Automated External Defibrillator (AED) Applied Prior to EMS | *X* | 0.00059809 | -0.0013304 | *X* | 0.00166463 | -0.0016709 |
| 1. Who First Defibrillated the Patient | *X* | -0.0002369 | -0.0005158 | *X* | 0.00058952 | 0.00030099 |
| 1. Did 911 Responder Perform CPR | *X* | 0.00903622 | -0.0657175 | *X* | 0.03799284 | -0.0044647 |
| 1. First Monitored Rhythm | *X* | 0.01380404 | -0.0756317 | *X* | 0.03738283 | -0.0079582 |
| 1. First Rhythm Type | *X* | 0.00022291 | -0.0015605 | *X* | 0.01542607 | -0.0043935 |
| 1. Sustained ROSC | *X* | 4.6103E-05 | -3.379E-05 | *X* | 0.00015782 | -4.947E-05 |
| 1. Field Targeted Temperature Management | *X* | 0.00093523 | -0.0030793 | *X* | 0.01530161 | -0.0037759 |
| 1. Advanced Airway Successfully Placed | *X* | 2.4906E-05 | -0.0012717 | *X* | 0.00287528 | -0.0011907 |
| 1. Advanced Airway Detail | *X* | 0.00038546 | -0.0045242 | *X* | 0.03636866 | -0.0087095 |
| 1. Were Drugs Administered | *X* | -0.0001418 | -9.537E-05 | *X* | 0.00054584 | 0.00010158 |
| 1. Vascular Access | *X* | 5.0447E-05 | -0.0026212 | *X* | 0.00176123 | -0.0003176 |
| 1. 2 Lead electrocardiogram | *X* | 0.00018367 | 0.00018062 | *X* | 0.00722455 | 0.00017855 |
| 1. Final Diagnosis Myocardial Infarction | *X* | 0.01651175 | -0.0650415 | *X* | 0.00574885 | -0.0006932 |
| 1. Coronary Angiography |  |  |  | *X* | 0.03944076 | -0.0104142 |

Table S3: Detailed Parameters for Each Model

| **Model** | **Grid Search Parameters** | **Key Model Parameters Selected** | |
| --- | --- | --- | --- |
|  |  | CA | CPC |
| Logistic Regression | Penalty={L1,L2}  C={0.5,1,2,3,4} | Penalty=L2  C=1 | Penalty=L2  C=1 |
| Decision Tree | Min Samples Split={2,4,8}  Min Samples Leaf={1,2,4,8} | Min Samples Split=2  Min Samples Leaf=1 | Min Samples Split=3  Min Samples Leaf=1 |
| Random Forest | Number Estimators={50,100,200,500,1000}  Max Depth={3,5,7} | Number Estimators=1000  Max Depth=7 | Number Estimators= 750  Max Depth=7 |
| k- Nearest Neighbor | k={1,3,5,7,9} | k=7 | k=7 |
| Gradient Boost | Max Depth={3,5,7}  Min Child Weight={3,5,7}  Number Estimators={50,100,200,500,1000} | Max Depth=7  Min Child Weight=5  Number Estimators=200 | Max Depth=7  Min Child Weight=5  Number Estimators=100 |
| XGBoost | Gamma={1,1.2,1.5,1.7}  Max Depth={3,5,7}  Min Child Weight={3,5,7}  Number Estimators={50,100,200,500,1000} | Gamma=1.5  Max Depth=5  Min Child Weight=5  Number Estimators=500 | Gamma=1.2  Max Depth=7  Min Child Weight=5  Number Estimators=500 |
| LightGBM | Colsample Bytree={0.55,0.65,0.75,0.85,1}  Learning Rate={0.01,0.001,0.05, 0.005}  Number Estimators={50,100,200,500,1000}  Number Leaves={4,8,16,32} | Colsample Bytree=0.65  Learning Rate=0.01  Number Estimators=100  Number Leaves=16 | Colsample Bytree=0.65  Learning Rate=0.001  Number Estimators=200  Number Leaves=8 |
| SVM | C={0.5,1,2,3,4}  Kernel={‘linear’,’rbf’,’poly’,’sigmond’} | C=2  Kernel=rbf | C=0.5  Kernel=sigmond |
| EFCN | Embedding Dim = {50}  Filter Size={32,64,128,256}  Kernel Size={3,5,8}  Activation={TanH, ReLU,} | Embedding Dim = 50  CNN Parameters  Filter Size = (64,128,64)  Kernel Size = (8,5,5)  Activation = ReLU | Embedding Dim = 50  CNN Parameters  Filter Size = (128,256,128)  Kernel Size = (8,5,3)  Activation = ReLU |
| Note: Model Parameters for models (besides EFCN) that are not listed are set as the Sklearn, XGBoost, or LightGBM Default  Note2: All random seeds for models are set to 0 | | | |

Table S4: Detailed modeling results on both the validation and testing sets

| **Model** | **Metric** | **Validation** | | **Testing** | |
| --- | --- | --- | --- | --- | --- |
|  |  | **CA** | **CPC** | **CA** | **CPC** |
| LightGBM | AUROC  [Confidence Inter.] | 0.7050  [0.62067, 0.78937] | 0.7462  [0.69240, 0.80016] | 0.72258  [0.68174, 0.76343] | 0.74411  [0.70807, 0.78014] |
|  | Accuracy | 0.8215 | 0.8713 | 0.84 | 0.865 |
|  | AUCPR | 0.59878 | 0.96002 | 0.66133 | 0.95312 |
|  | F1 Score | 0.54736 | 0.92191 | 0.58260 | 0.91743 |
|  | MCC | 0.44056 | 0.57796 | 0.49211 | 0.56588 |
|  | BLS | 0.13896 | 0.09803 | 0.11613 | 0.10779 |
|  | Confusion  Matrix | 172 \| 17  26 \| 26 | 27 \| 24  7 \| 183 | 437 \| 33  63 \| 67 | 69 \| 61  20 \| 450 |
| Random  Forest | AUROC  [Confidence Inter.] | 0.6641  [0.58214, 0.74620] | 0.6437  [0.57904, 0.70836] | 0.71202  [0.67049, 0.75356] | 0.66350  [0.62724, 0.69976] |
|  | Accuracy | 0.8340 | 0.8340 | 0.85833 | 0.84333 |
|  | AUCPR | 0.59764 | 0.94560 | 0.68603 | 0.95212 |
|  | F1 Score | 0.48717 | 0.90243 | 0.58128 | 0.90748 |
|  | MCC | 0.43537 | 0.41623 | 0.53441 | 0.47074 |
|  | BLS | 0.13253 | 0.11460 | 0.11378 | 0.11294 |
|  | Confusion  Matrix | 182 \| 7  33 \| 19 | 16 \| 35  5 \| 185 | 456 \| 14  71 \| 59 | 45 \| 85  9 \| 461 |
| XGBoost | AUROC  [Confidence Inter.] | 0.6639  [0.58222, 0.74561] | 0.7561  [0.69900, 0.81317] | 0.72986  [0.68990, 0.76983] | 0.72765  [0.69134, 0.76397] |
|  | Accuracy | 0.8008 | 0.8755 | 0.83833 | 0.85666 |
|  | AUCPR | 0.55867 | 0.95571 | 0.62864 | 0.95227 |
|  | F1 Score | 0.47826 | 0.92424 | 0.59071 | 0.91260 |
|  | MCC | 0.36246 | 0.59376 | 0.49478 | 0.53531 |
|  | BLS | 0.64895 | 0.10339 | 0.12249 | 0.10633 |
|  | Confusion  Matrix | 171 \| 18  30 \| 22 | 28 \| 23  7 \| 183 | 433 \| 37  60 \| 70 | 65 \| 65  21 \| 449 |
| Gradient  Boost | AUROC  [Confidence Inter.] | 0.6619  [0.58044, 0.74352] | 0.71279  [0.64644, 0.77914] | 0.73461  [0.69371, 0.77551] | 0.68396  [0.64280, 0.72511] |
|  | Accuracy | 0.7759 | 0.82987 | 0.82833 | 0.81 |
|  | AUCPR | 0.53885 | 0.93870 | 0.63189 | 0.93502 |
|  | F1 Score | 0.47058 | 0.89460 | 0.58964 | 0.88198 |
|  | MCC | 0.32865 | 0.45825 | 0.48177 | 0.40042 |
|  | BLS | 0.16302 | 0.14753 | 0.13295 | 0.15902 |
|  | Confusion  Matrix | 163 \| 26  28 \| 24 | 26 \| 25  16 \| 174 | 423 \| 47  56 \| 74 | 60 \| 70  44 \| 426 |
| Decision Tree | AUROC  [Confidence Inter.] | 0.6415  [0.56686, 0.71620] | 0.66615  [0.59215, 0.74014] | 0.68625  [0.64006, 0.73244] | 0.67086  [0.62456, 0.71717] |
|  | Accuracy | 0.7219 | 0.76763 | 0.77 | 0.76333 |
|  | AUCPR | 0.49797 | 0.91340 | 0.55571 | 0.91184 |
|  | F1 Score | 0.43697 | 0.85106 | 0.50359 | 0.84665 |
|  | MCC | 0.25990 | 0.32341 | 0.35600 | 0.32964 |
|  | BLS | 0.27800 | 0.23236 | 0.23 | 0.23666 |
|  | Confusion  Matrix | 148 \| 41  26 \| 26 | 25 \| 26  30 \| 160 | 392 \| 78  60 \| 70 | 66 \| 64  78 \| 392 |
| k-Nearest  Neighbor | AUROC  [Confidence Inter.] | 0.6781  [0.59607 0.76015] | 0.7077  [0.64189, 0.77368] | 0.70270  [0.65527, 0.75012] | 0.68363  [0.63104, 0.73621] |
|  | Accuracy | 0.8340 | 0.8672 | 0.835 | 0.84 |
|  | AUCPR | 0.54892 | 0.94747 | 0.64388 | 0.91140 |
|  | F1 Score | 0.51219 | 0.92118 | 0.55203 | 0.90380 |
|  | MCC | 0.44387 | 0.55670 | 0.46561 | 0.46560 |
|  | BLS | 0.14243 | 0.10551 | 0.12428 | 0.13031 |
|  | Confusion  Matrix | 180 \| 9  31 \| 21 | 22 \| 29  3 \| 187 | 440 \| 30  69 \| 61 | 53 \| 77  19 \| 451 |
| Logistic  Regression | AUROC  [Confidence Inter.] | 0.6937  [0.61492, 0.77253] | 0.7417  [0.68797, 0.79550] | 0.74582  [0.70488, 0.78676] | 0.72765  [0.69280, 0.76251] |
|  | Accuracy | 0.8257 | 0.8755 | 0.86333 | 0.85666 |
|  | AUCPR | 0.58878 | 0.96020 | 0.68543 | 0.95523 |
|  | F1 Score | 0.53333 | 0.925 | 0.63063 | 0.91260 |
|  | MCC | 0.43734 | 0.58987 | 0.56215 | 0.53531 |
|  | BLS | 0.13529 | 0.09637 | 0.10927 | 0.10714 |
|  | Confusion  Matrix | 175 \| 14  28 \| 24 | 26 \| 25  5 \| 185 | 448 \| 22  60 \| 70 | 65 \| 65  21 \| 449 |
| Support Vector Machine | AUROC  [Confidence Inter.] | 0.67282  [0.59595, 0.74969] | 0.62936  [0.57175, 0.68696] | 0.72831  [0.68530, 0.77132] | 0.62225  [0.58435, 0.66016] |
|  | Accuracy | 0.82572 | 0.83402 | 0.85333 | 0.82666 |
|  | AUCPR | 0.58863 | 0.95520 | 0.68530 | 0.94982 |
|  | F1 Score | 0.50000 | 0.90338 | 0.6 | 0.89883 |
|  | MCC | 0.41900 | 0.41270 | 0.52683 | 0.39481 |
|  | BLS | 0.13167 | 0.10708 | 0.11089 | 0.10961 |
|  | Confusion  Matrix | 178 \| 11  31 \| 21 | 14 \| 37  3 \| 187 | 446 \| 24  64 \| 66 | 34 \| 96  8 \| 462 |
| EFCN | AUROC  [Confidence Inter.] | 0.8836  [0.85998, 0.90730] | 0.9272  [0.91315, 0.94143] | 0.90793  [0.90066, 0.91521] | 0.89672  [0.88384, 0.90960] |
|  | Accuracy | 0.9377 | 0.9419 | 0.95166 | 0.91666 |
|  | AUCPR | 0.91353 | 0.99474 | 0.95727 | 0.99162 |
|  | F1 Score | 0.84536 | 0.96276 | 0.88163 | 0.94600 |
|  | MCC | 0.80995 | 0.83173 | 0.85393 | 0.76538 |
|  | BLS | 0.08552 | 0.06691 | 0.07277 | 0.06825 |
|  | Confusion  Matrix | 185 \| 4  11 \| 41 | 46 \| 5  9 \| 181 | 463 \| 7  22 \| 108 | 122 \| 18  32 \| 438 |
| **AUCPR:** Area Under the Precision-Recall Curve  **MCC:** Matthews correlation coefficient  **BLS:** Brier score loss | | | | | |
